# The Prevention of Viral Infections: The Role of Intestinal Microbiota and Nutritional Factors

**DOI:** 10.3390/nu16152445

**Published:** 2024-07-27

**Authors:** Annamaria Altomare, Marta Giovanetti, Francesca Baldaro, Massimo Ciccozzi, Michele Cicala, Michele Pier Luca Guarino

**Affiliations:** 1 Department of Sciences and Technologies for Sustainable Development and One Health, Università Campus Bio-Medico di Roma, 00128 Rome, Italy; a.altomare@unicampus.it (A.A.); m.giovanetti@unicampus.it (M.G.); 2 Unit of Gastroenterology, Università Campus Bio-Medico di Roma, 00128 Rome, Italy; francesca.baldaro@unicampus.it (F.B.); m.guarino@unicampus.it (M.P.L.G.); 3 Instituto Rene Rachou, Fundação Oswaldo Cruz, Belo Horizonte 30190-002, Brazil; 4 Climate Amplified Diseases and Epidemics (CLIMADE), Bairro Floresta 31110-370, Brazil; 5 Unit of Medical Statistics and Molecular Epidemiology, Università Campus Bio-Medico di Roma, 00128 Rome, Italy; m.ciccozzi@unicampus.it; 6 Unit of Gastroenterology and Digestive Endoscopy, Fondazione Policlinico Campus Bio-Medico di Roma, 00128 Rome, Italy

**Keywords:** viral infection, nutrition, microbiota, vitamins, probiotics

## Abstract

Viral infections pose significant global challenges due to their rapid transmissibility. Therefore, preventing and treating these infections promptly is crucial to curbing their spread. This review focuses on the vital link between nutrition and viral infections, underscoring how dietary factors influence immune system modulation. Malnutrition, characterized by deficiencies in essential nutrients such as vitamins A, C, D, E, and zinc, can impair the immune system, thereby increasing vulnerability to viral infections and potentially leading to more severe health outcomes that complicate recovery. Additionally, emerging evidence highlights the role of commensal microbiota in immune regulation, which can affect hosts’ susceptibility to infections. Specific dietary components, including bioactive compounds, vitamins, and probiotics, can beneficially modify gut microbiota, thus enhancing immune response and offering protection against viral infections. This review aims to elucidate the mechanisms by which dietary adjustments and gut microbiota impact the pathogenesis of viral infections, with a particular focus on strengthening the immune system.

## 1. Introduction

Nutrition plays a crucial role in modulating the immune system, and the gut microbiota is a key mediator in this process. Diet influences the composition and function of gut microbiota, which in turn affects the body’s ability to respond to viral infections [1]. Specific dietary components such as fibers, vitamins, and polyphenols can enhance gut health. High-fiber diets promote beneficial bacteria that produce short-chain fatty acids (SCFAs), which have anti-inflammatory properties and support immune function [2]. 

Indeed, a healthy gut can enhance immune responses in the lungs, potentially offering protection against respiratory viral infections, as it has been demonstrated that the gut microbiota can influence respiratory health through the gut–lung axis [3]. It is well defined that dysbiosis, or an imbalance in gut microbiota, can exacerbate viral infections by weakening the immune response [3,4]. This can lead to more severe outcomes in infections such as influenza and COVID-19, as recent evidence has highlighted [5,6]. 

Moreover, deficiencies in essential nutrients like vitamins A, D, C, and zinc can impair immune function and alter gut microbiota composition, making individuals more susceptible to infections, including viral ones [7].

All the recent evidence suggests that modulating the intestinal microbiota, starting with a healthy diet and specific supplements, could be an interesting strategy to contrast emerging viral infections in the coming years [8]. 

For this reason, the present review aims to highlight the mechanisms of strengthening the immune system to prevent viral infections with particular attention to the role of gut microbiota and dietary habits. 

After a brief introduction to the most current evidence regarding the spread of viral infections worldwide, the structure of the review is divided into two chapters. Section 1 explores the role of the intestinal microbiota in promoting immune defenses and possibly preventing and/or modulating the spread of viral infections through its modulation using probiotics. Section 2 summarizes the role of different dietary patterns on gut microbiota composition responsible for different effects on gut microbiota health. Finally, the last paragraph of Section 2 describes the current evidence about specific dietary components with antiviral properties. 

## 2. Interplay of Viral Infections and Microbiota

Given the profound impact of infectious diseases on global health, particularly highlighted by recent pandemics [9,10,11], it is crucial to thoroughly explore the interactions between viral infections and the indigenous microbiota. These interactions are not only complex but also play a significant role in determining the course and outcome of infections. Understanding how the microbiota influences viral pathogenesis and the host immune response can provide valuable insights into developing more effective treatments and preventive strategies for infectious diseases [12,13,14]. 

The spread of viruses, such as adenovirus, influenza, and SARS-CoV-2; gastrointestinal viruses like norovirus and rotavirus; and others affecting the liver and cervix, such as hepatitis and HPV, begins with their interaction with specific host–cell receptors. Their lifecycle involves integration into the host–cell genome for some, like HIV and hepatitis viruses, while others are transient, eliciting a rapid immune response [15,16].

Furthermore, the continuous immune stimulation by microbial factors contributes significantly to antiviral defenses. This growing understanding has led to explorations of how “controlling microbiota” strategies might protect against viral infections [17]. Indeed, the interaction between host cells and microbiota on epithelial surfaces is crucial for understanding viral pathogenesis. This crosstalk affects viral gene expression and can influence the clinical outcomes of viral infections. It also highlights the systemic rather than local effects of the microbiota, suggesting a “gut–multiple organ” axis where the microbiota could govern the clinical outcomes during infections. In the context of viral infections, integrating the study of nutrition and intestinal microbiota becomes vital [18]. 

The microbiota’s composition and health are influenced by diet, which can affect the body’s response to viral infections. Modulating the intestinal microbiota through dietary changes, supplemented by lifestyle factors such as moderate exercise and stress management, may enhance resistance to viral infections and mitigate their severity. This approach not only helps in managing infections like those caused by respiratory and gastrointestinal viruses but also in preventing severe outcomes following infections [19]. 

Therefore, this review extends beyond traditional virology and molecular biology to include nutritional and microbial perspectives. By examining the interactions between diet, microbiota, and viral infections, we can gain insights into novel preventive and therapeutic strategies that leverage the microbiota’s potential to influence viral pathogenesis and outcomes. This holistic approach, considering both microbial and nutritional factors, provides a comprehensive framework for understanding and combating viral diseases across various tissues and organ systems.

## 3. The Role of Intestinal Microbiota in Promoting Immune Defenses

### 3.1. Intestinal Microbiota Development and Immune System

Intestinal microbiota represents a complex dynamic system composed of a heterogeneous community of microorganisms, which exert several essential functions for human health [20]. The human gut microbiota, enriched by more than 250 species of bacteria, fungi, viruses, and archaea, changes throughout human life, and it is highly variable among individuals, depending on numerous factors such as age; diet; drugs, including antibiotics; and other environmental factors [21]. 

In the literature, the importance of the gut microbiota in the development of the immune system in the early stages of life is well known; in fact, germ-free mice display significant reductions in both immunoglobulin A (IgA) and T helper 17 (Th17) [22]. Key interactions include the production of SCFAs that regulate immune cell function, the stimulation of pattern recognition receptors (PRRs) such as Toll-like receptors (TLRs), and the induction of regulatory T cells (Tregs) that maintain immune homeostasis [23]. In particular, gut microbiota ferments indigestible dietary fibers, producing SCFAs such as acetate, propionate, and butyrate, the most common microbial metabolites in the colic lumen. SCFA inhibits the NF-kB signaling pathway with anti-inflammatory effects by blocking the transcription of several pro-inflammatory cytokines such as TNF-α, TNF-β, IL-1β, IL-3, IL-5, IL-12, and IL-18 [22]. In addition, SCFA plays a crucial role in training the immune system to distinguish between commensal and pathogenic microorganisms by stimulating the expression on the innate immune cells of TLRs, a particular type of pattern recognition receptor (PRR) involved in the recognition of pathogen association molecular patterns (PAMPs) such as LPS [23]. SCFA stimulates the differentiation of naive T cells into effector T cells such as Th1 and Th17 effector T cells, which are closely involved in activating the adaptive response [23].

PRRs are highly expressed in intestinal epithelial cells (IECs), macrophages, and intestinal dendritic cells (DCs). PRRs identify microbe- or pathogen-associated molecular patterns (MAMPs or PAMPs) on pathogens and commensals alike [24,25]. Once a microbe is identified or breaches the epithelium, the immune system mounts a targeted response against it [26]. Pattern recognition receptors (PRRs) activate various intracellular signaling pathways after PAMPs’ recognition. In particular, these pathways involve chains of ligands, transcription factors, and kinases to signal infection presence in the host and induce changes in gene expression. The activation of these pathways affects levels of pro-inflammatory and antimicrobial cytokines, chemokines, and immunoreceptors. Reduced levels of pro-inflammatory cytokines like IL-23, IL-12, and IL-8 contribute to protective effects by decreasing the production of anti-inflammatory cytokines such as IL-10 by regulatory T cells [27]. In this context, another example is represented by the activation of TLR-5 by the interaction with flagellin, a bacterial protein involved in pathogen mobility; the activation of TLR-5, expressed on B-cells, is related to their differentiation into IgA-producing cells [25]. In addition to the known interactions between microbiota and innate immunity, several studies document mutualism between microbiota and adaptive immunity. SCFA stimulates the differentiation of naive T cells into effector T cells such as Th1 and Th17 effector T cells, which are closely involved in activating the adaptive response [23]. To reinforce this mutualism relationship, in a recent study, it was found that in germ-free mice, no differentiation of a subset of CD4 T-regulatory cells occurs due to the inability to produce SCFAs [28].

Another example of the interaction between microbiota and adaptive immunity concerns CD8+ T cells, which, in response to interaction with antigen-presenting cells (APCs), undergo maturation. In germ-free mice following interaction with APC cells, maturation does not occur because the absence of SCFA from the microbiota is necessary for their memory potential to develop [29].

In addition, a recent study on mice demonstrated that fecal IgA levels significantly increased following prebiotic treatment. These findings suggest that the gut microbiome plays a crucial role in maintaining intestinal mucosal immune balance [30]. Gut dysbiosis is a condition characterized by changes in gut bacterial composition and functional capabilities that result in negative impacts on host health [31]. Certain commensal bacteria such as Bifidobacterium inhibit the growth of opportunistic pathogens like Escherichia Coli by producing short-chain fatty acids (SCFAs) during lactose fermentation, which change the intestinal pH [32].

### 3.2. Dysbiosis and Viral Infections

Gut dysbiosis is a condition characterized by changes in gut bacterial composition and functional capabilities that result in negative impacts on host health [32]. Certain commensal bacteria such as Bifidobacterium inhibit the growth of opportunistic pathogens like Escherichia Coli by producing SCFAs during lactose fermentation, which change the intestinal pH [17].

**Figure 1:**
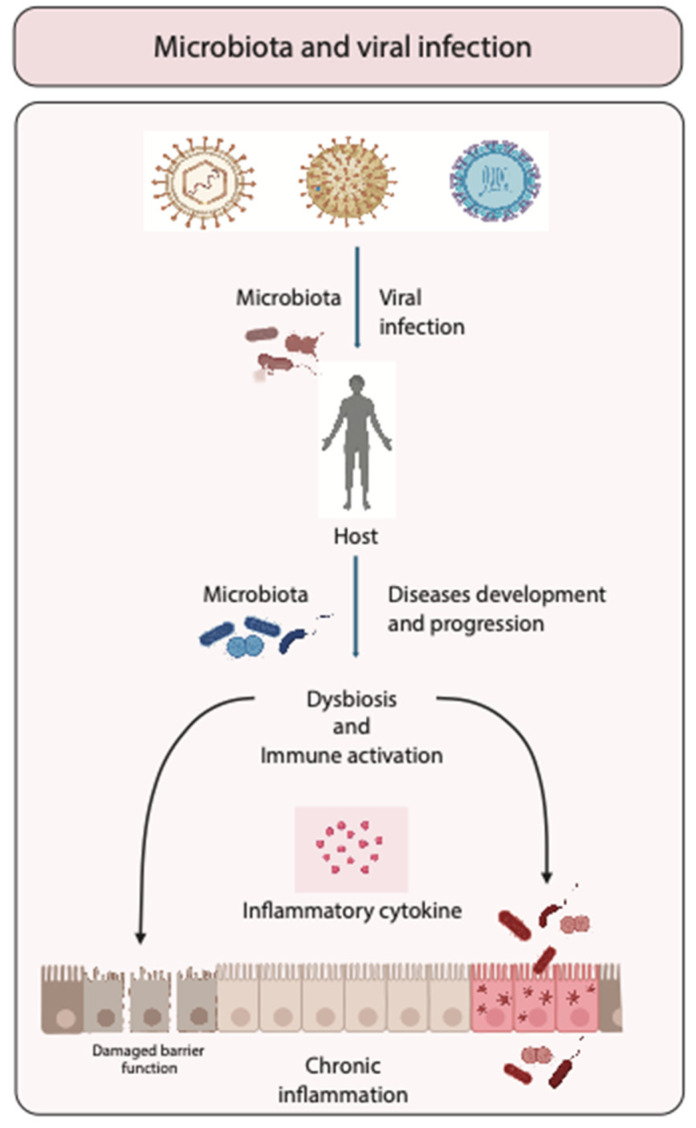
Role of microbiota in viral infections. Schematic representation of the interaction between microbiota and viruses during infection, illustrating how microbiota can either enhance or suppress viral infections. The figure emphasizes the impact of variations in the composition of the intestinal microbiota on disease progression, potentially leading to dysbiosis and immune activation that might potentially enhance chronic inflammation.

The intricate interplay between the human microbiota and viral infections presents a critical avenue of study in modern medical science. Dysbiosishas been identified as a significant factor influencing both susceptibility to and the severity of viral infections. The mechanisms through which dysbiosis can impact viral pathogenesis are multifaceted, involving alterations in barrier integrity, immune modulation, and even direct effects on viral replication (Figure 1). 

Dysbiosis typically results from factors such as antibiotic use, dietary changes, or underlying illnesses and can lead to compromised mucosal barriers [32]. These barriers, particularly in the respiratory and gastrointestinal tracts, serve as the body’s first line of defense against pathogens. When their integrity is compromised due to an imbalanced microbiota, viruses find an easier pathway to enter the host. For instance, influenza and other respiratory viruses can exploit weakened mucosal defenses to establish infections more readily. Research suggests that individuals with altered gut microbiota experience more severe respiratory infections due to this compromised barrier function [33]. 

Beyond physical barriers, the microbiota plays a crucial role in shaping the immune response. An imbalanced microbial environment can lead to inappropriate immune reactions, where the body either fails to mount a sufficient response to the viral invader or launches an overly aggressive response that can lead to tissue damage. In the case of HIV, dysbiosis in the gastrointestinal tract has been linked to increased levels of systemic inflammation and faster disease progression due to enhanced microbial translocation across weakened intestinal barriers [18]. The altered immune landscape not only facilitates increased HIV replication but also exacerbates the immune system’s decline. Furthermore, the microbiota can influence viral replication directly. Certain microbial components and metabolites have been shown to either promote or inhibit viral replication. For example, some gut bacteria produce short-chain fatty acids that can have antiviral effects, potentially reducing the replication rate of certain pathogens. Conversely, some viruses may utilize metabolites produced by bacteria to enhance their own replication processes, a phenomenon that highlights the complex interactions at play within the microbiota–virus dynamic [34]. 

For instance, targeting specific bacteria that exacerbate viral replication could become a novel approach in viral infection management. The exploration of the microbiota’s role in viral infections extends beyond treatment into prevention. The potential to manipulate the microbiota to bolster the body’s defenses against upcoming viral threats could revolutionize how we approach viral epidemics. Research in this area continues to expand, revealing new insights into how the complex network of microorganisms that inhabit our bodies interacts with viral pathogens. These studies underscore the potential of microbiota-focused strategies in managing and preventing viral diseases, highlighting the need for further research to fully harness this potential.

### 3.3. Intestinal Microbiota Modulation and Prevention of Viral Infections: Role of Probiotics

Given the significant impact of dysbiosis on viral pathogenesis, therapeutic strategies aimed at modifying the microbiota are being explored. Probiotics, which aim to restore or maintain a healthy microbiota, have shown the potential to mitigate the severity of viral infections. Studies have indicated that probiotics may enhance immune function and reduce the duration and severity of viral infections, such as influenza [35]. Additionally, the specific microbial compositions associated with different viral infections are becoming key targets for therapeutic interventions [36]. Understanding these relationships can help in developing personalized microbiota management strategies that enhance the effectiveness of traditional antiviral therapies. 

The intestinal microbiota plays a crucial role in modulating the immune system and preventing viral infections. A balanced gut microbiota supports the integrity of the intestinal barrier, preventing the translocation of pathogens and toxins. It also promotes the production of short-chain fatty acids (SCFAs) like butyrate, which have anti-inflammatory properties and enhance the function of immune cells [37,38]. 

Specific probiotics and prebiotics can beneficially alter the gut microbiota composition, enhancing the host’s immune response. Studies have shown that certain probiotic strains can increase the production of antiviral cytokines and improve the activity of natural killer cells and T cells [39]. For instance, Lactobacillus and Bifidobacterium species have been linked to increased resistance against viral infections such as influenza and norovirus [40,41]. Probiotics are most commonly bacterial species from the Lactobacillus and Bifidobacterium genera, and their use as treatment for conditions with an inflammatory component is widely documented, since probiotics can induce both pro- and anti-inflammatory responses [41]. The preventive effects of *Lactobacillus rhamnosus GG* (LGG) on experimental rhinovirus infections in healthy volunteers were studied. Participants (n. 59) were randomized to ingest LGG for six weeks, receiving 100 mL of fruit juice supplemented with 10 × 9 cfu, live or heat-inactivated (by spray-drying), before being intranasally inoculated with rhinovirus. The study assessed infection rates, occurrence, and the severity of cold symptoms. The results showed that the LGG group experienced lower frequencies and severities of cold symptoms, as well as fewer rhinovirus infections compared to the control group, although these differences were not statistically significant due to the small sample size [42]. Even if the exact mechanisms behind the immunomodulatory effects of probiotics are not fully understood, LGG has been shown to modulate both innate and adaptive immune responses. In another study, LGG was given for four weeks to children with gastroenteritis caused by rotavirus or Cryptosporidium species [43]. A significant increase in serum immunoglobulin IgG levels was observed post-intervention in children with rotavirus-induced diarrhea who received LGG [43]. Among children with cryptosporidial diarrhea, those treated with LGG showed a significant improvement in intestinal permeability [44]. In 2015, Jespersen et al. showed that *Lactobacillus casei 431′* administration was related to the reduction in the duration of upper respiratory symptoms, although it showed no effect on the immune response to influenza vaccination in healthy adults [45]. Similarly, Rizzardini et al. showed a significantly greater implementation from baseline in the titers of vaccine-specific IgG, IgG1, and IgG3 in plasma as well as that of vaccine-specific secretory IgA in saliva in both probiotic groups, as compared with the control group [46]. In 2010, Grandy et al. showed that rehydration therapy associated with the oral administration of *Saccharomyces boulardii* was significantly associated with the reduction in the duration of diarrhea in acute rotavirus gastroenteritis children in Bolivia, compared with control rehydration alone [47]. Similarly, Erdoğan et al. reported that oral rehydration therapy administered with *Bifidobacterium lactis B94* significantly shortened the diarrheal period in acute rotavirus gastroenteritis children compared with control oral rehydration alone [48].

Although the precise mechanisms by which microbiota modulation can be employed in the prevention and treatment of disease remain unclear, there is a growing body of evidence suggesting that this approach may offer a promising avenue for future research.

## 4. Dietary Habits, Gut Microbiota Modulation, and Prevention of Viral Infections

### 4.1. Dietary Habits and Gut Microbiota Modulation

It is well known that diet, together with other factors such as physical activity and an equilibrated sleep–wake rhythm, plays a strategic role in promoting health and preventing non-communicable diseases [49]. Among the mechanisms involved in this beneficial effect, the modulation of intestinal microbiota is one of the most studied in recent years [50]. Evidence from the last few decades highlights that a plant-based eating pattern rich in cereals, legumes, vegetables, fruits, olive oil, and nuts—typical of countries such as Greece, Spain, and Italy and called the Mediterranean Diet (MD)—is associated with the promotion of an equilibrated microbiota composition, which is associated with a beneficial effect on host health [51]. Particularly, it has been demonstrated that the MD dietary pattern positively influences gut microbiota by increasing beneficial bacteria like Bifidobacteria and Lactobacilli, which are associated with anti-inflammatory effects and improved metabolic health [51]. 

The intestinal microbiota is essential for several metabolic and immune functions, thanks to its ability to recover energy from food through the synthesis of specific digestive enzymes [52,53], providing hosts with vitamins (like thiamine, folate, biotin, riboflavin, and pantothenic acid), and maintaining the integrity of the intestinal epithelial barrier [54,55,56]. It is also crucial for the development of the functional maturation of the gut immune system and to contrast exogenous pathogens by a competitive mechanism [56]. 

The MD is rich in fibers with prebiotic activity; polyunsaturated fatty acids with anti-inflammatory properties; and several bioactive compounds with antioxidative properties such as polyphenols, vitamins, and minerals in adequate amounts [50]. Most of these nutrients are also able to promote the growth of beneficial bacteria, increasing a virtuous cycle of strengthening the metabolic and defensive action of the intestinal microbiota [51]. 

Moreover, much evidence indicates that plant-based diets, including vegetarian and vegan diets, also enhance microbial diversity and promote a higher abundance of fiber-degrading bacteria, leading to the increased production of short-chain fatty acids (SCFAs) that support gut health and reduce inflammation [2].

Conversely, it is well demonstrated that a high-fat diet (HFD) is responsible for the generation of intestinal dysbiosis characterized by an increased Firmicutes/Bacteroidetes ratio with a significant increase in *Dorea*, *Ruminocuccus*, *Erysipelotrichales*, *Bacilli*, and *Clostridiales* and a significant reduction in Prevotellaceae and Rikenellaceae, belonging to the Bacteroidetes phylum, which exerts important anti-inflammatory activities promoting gut barrier integrity [57,58,59,60]. In animal and human models, obesity and HFD has been correlated with increased endotoxemia associated with altered intestinal permeability and the activation of several pro-inflammatory pathways [61,62,63]. It has been demonstrated that the increase in Firmicutes and Actinobacteria, typical of an HFD, is positively correlated with the activation of colonic macrophages and the production of pro-inflammatory cytokines, such as TNF-α, IL-1β, and IL-6 [62]. Furthermore, the higher amount of animal protein in the HFD of Western countries determines a significant increase in bile-tolerant microorganisms such as *Alistipes*, *Bilophila*, and *Bacteroides* and a decreased level of Firmicutes that metabolize dietary plant polysaccharides such as *Roseburia*, *Reubacterium rectale*, and *Ruminococcus bromii* [63,64,65]. Another negative modulator of the HFD for the intestinal microbiota seems to be the higher presence of food additives which can promote an imbalance in the microbiota composition dysbiosis associated with altered mucosal permeability and the genesis of inflammatory processes [66]. 

Conflicting data are present about changes in gut microbiota composition due to a gluten-free diet (GFD); actually, a GFD may decrease microbial diversity, which is generally considered less favorable for gut health. This reduction in diversity might be due to the lower intake of dietary fibers found in gluten-containing grains [67]. It has been shown that individuals on a GFD often exhibit reduced levels of beneficial Bifidobacteria and an increased proportion of potentially pathogenic bacteria like Enterobacteriaceae [68]. At the same time, some evidence suggests that a GFD improves metabolic parameters in individuals with celiac disease or non-celiac gluten sensitivity, potentially through the modulation of gut microbiota [69]. Interestingly, GFD, reducing gut inflammation in those with celiac disease by removing the inflammatory trigger (gluten), can help restore a healthier gut environment and microbiota balance [70]. However, the long-term impact on healthy individuals remains unclear.

**Figure 2:**
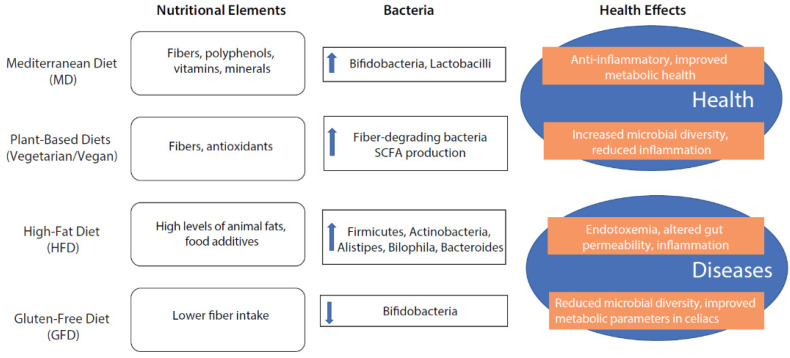
The effect of different nutritional patterns on intestinal microbiota composition promoting health or disease.

In summary, different dietary patterns have nutritional elements with properties to modulate the gut microbiota, thus promoting health or disease (Figure 2). 

### 4.2. Dietary Components with Antiviral Properties

**Figure 3:**
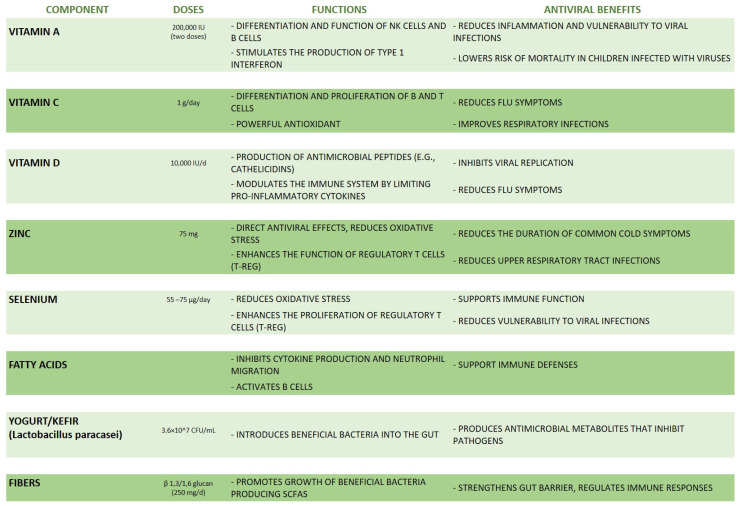
A summary of the main dietary components with antiviral properties.

A well-balanced diet plays a crucial role in bolstering the immune system and preventing viral infections, particularly through its impact on gut microbiota. Indeed, dietary interventions that support microbiota diversity and stability can strengthen the immune response and potentially reduce the impact of viral pathogens. In this paragraph, the current evidence about specific dietary components with antiviral properties is described, underlying the importance of a rich and complete diet as a powerful weapon for promoting health and preventing viral infections (Figure 3).

#### 4.2.1. Vitamins

Micronutrients such as vitamins and nutritionally essential minerals support every phase of the immune process. Vitamin or mineral deficiencies are involved both in innate and adaptive immunity, causing immunosuppression and viral infection vulnerability [7,71,72]. 

Nutrients such as vitamins A, C, D, and E, as well as minerals like zinc and selenium, are vital for supporting immune function, as summarized in Figure 3. For example, vitamin A stimulates the differentiation and function of NK cells’ and B cells’ growth and proliferation [71]. 

Vitamin A is a class of fat-soluble vitamins essential for growth, vision, and immune response. Carotenoids like β-carotene are provitamins A that are converted into retinol and retinoic acid, the most bioactive form of vitamin A, in the intestine and absorbed by the body [73]. In a recent study, it was demonstrated that a lack of vitamin A results in heightened inflammation and a greater vulnerability to viral infections [74]. Additionally, Sarohan explained that during inflammatory diseases such as COVID-19, a common issue is the depletion of retinoic acid, which leads to the breakdown of the immune system by hindering the production of type 1 interferon [74]. Furthermore, it has been observed that vitamin A administration has beneficial effects in children infected with viruses, especially in measles, in which two doses of vitamin A (200,000 international units (IUs) on consecutive days) reduced the mortality in children aged less than two years and pneumonia-specific mortality [75]. Moreover, the mean durations of pneumonia and other systemic symptoms, such as diarrhea and fever, were shorter, and the mean number of hospitalization days was lower [75]. Similarly, vitamin D is a fat-soluble vitamin derived from the liver conversion of 7-dehydrocholesterol and crucial in gut calcium absorption and in the production of several antimicrobial peptides such as cathelicidins, which act as potent antiviral agents [76]. In 2006, Klotman and Chang showed that cathelicidins, produced mainly by leukocytes and epithelial cells, possess chemotactic activity that inhibits viral replication [77]. Interestingly, vitamin D also modulates the immune system through various mechanisms such as the production of antimicrobial peptides or by limiting the cytokine pro-inflammatory production and inducing T reg cells’ differentiation [72]. Although current evidence is insufficient, it appears that a vitamin D deficiency has been associated with increased susceptibility to viral infections. The studies conducted so far suggest increasing the concentrations of 25(OH)D indicated above 40–60 ng/mL (100–150 nmol/L) through the administration of 10,000 IU/day of vitamin D3 for a few weeks.

Similarly, vitamin C, a water-soluble vitamin particularly present in citrus fruits with powerful antioxidant properties, enhances the differentiation and proliferation of B and T cells [72]. In 2019, Dobrange et al. showed that vitamin C boosts the immune system against various viral and bacterial infections [78], while in 2020, Gasmi et al. showed the beneficial role of vitamin C during upper respiratory infections; in fact, they showed that the regular administration of high vitamin C doses before or after the appearance of flu symptoms could prevent and relieve the flu symptoms in the test population in comparison to the control group [78]. 

#### 4.2.2. Minerals

Minerals such as zinc and selenium have direct antiviral effects by decreasing oxidative stress and enhancing the proliferation of T-reg cells [79]. In particular, the interaction between TLRs and antigens is followed by a rapid and transient zinc influx which was shown to reduce the production of type I IFNs. The role of the zinc influx in this context remains undefined but seems to prevent excessive IFN production [72]. Zinc supplementation has been shown to reduce upper respiratory tract infections, including pneumonia, rhinovirus infection, and common cold viruses such as influenza [80]. In particular, oral zinc supplementation at a dosage of at least 75 mg per day, taken continuously for at least five days and within three days of the onset of cold symptoms, was associated with a reduction in the duration of any symptoms. A daily dose of 75 mg of zinc has been found to reduce the duration of common cold symptoms by two days [81]. Similarly, selenium (Se), an essential trace element for humans, has a well-established history of decreasing the incidence and severity of viral infections. Selenium deficiency is associated with increased susceptibility to viral and bacterial infections due to its role in viral expression and immune function mediated by selenoproteins which are redox agents. Adding Se to the diet has been shown to improve immunity against viral infections [82]. Particularly, selenium supplementation modulates the immune response by stimulating T-cell proliferation and NK-cell activity. This stimulation seems to be stronger from inadequate to adequate selenium status at baseline, while the benefits of increasing an adequate selenium level to supra-nutritional levels are less clear [83]. The recommended US daily dose for adults is 55 μg/day [84], but in some countries, daily recommended doses are higher due to a lower average selenium status in these countries; for example, in the UK, the recommended daily Se dose is 60 μg/day for adult women and 75 μg/day for adult men [85]. Hoffmann et al. highlight the impact of selenium (Se) supplementation on immune function in mice, particularly through the modulation of CD4+ T-cell responses. Mice were fed diets with low (0.08 mg/kg), medium (0.25 mg/kg), or high (1.0 mg/kg) selenium content for 8 weeks and after the dietary intervention were challenged with a peptide/adjuvant to stimulate an immune response. Antigen-specific CD4+ T-cell responses were significantly higher in the high Se group compared to the low and medium Se groups. This suggests that higher selenium intake enhances the ability of CD4+ T cells to respond to antigens [86].

#### 4.2.3. Fibers

A diet rich in fiber from fruits, vegetables, and whole grains promotes the growth of beneficial gut bacteria. Before being fermented by colon microbes, inulins, pectins, and β-galactomannan mediate the tight junction’s protein assembly, supporting the functional gut epithelial barrier. The gut bacteria, especially *Clostridium*, *Bacteroides*, *Bifidobacterium*, *Prevotella*, and *Ruminococcus*, in turn, produce short-chain fatty acids (SCFAs) which, as potent immunomodulators, strengthen the gut barrier and regulate immune responses [87]. Fatty acids play a role in reinforcing immunity defenses by inhibiting both cytokine production and neutrophil migration and activating B cells [73]. Particularly, non-digestible polysaccharides (NDPs) are a diverse group of molecules found in various foods including ß-glucans, pectins, resistant starch, arabinoxylans, and other types derived from sources such as plants (e.g., ginseng, carrot, oat), fungi (e.g., Saccharomyces cerevisiae, Lentinula edodes), and bacteria (e.g., *Alcaligenes faecalis*). The pivotal role of NDPs in supporting health and immune activity is well known [87,88]. Clinical research has also supported these findings, with double-blind placebo-controlled studies showing that the oral consumption of certain NDPs, like β-glucans, can decrease the incidence and duration of upper respiratory tract infections (URTIs) in elderly individuals. Particularly, Fuller et al. demonstrated that the number of medically confirmed upper respiratory tract infections (URTIs) and the number of symptom days was significantly reduced after the administration of once-daily β-1,3/1,6 glucan (Wellmune 250 mg/d) versus a placebo capsule (n. 50) over 90 days during the winter period in community-dwelling adults (n. 50) aged 50 to 70 years old. Larger studies are needed to confirm the benefits of β-1,3/1,6 glucan on URTIs in this older population [89].

#### 4.2.4. Food with Probiotic Properties

Probiotics found in fermented food such as yogurt and kefir directly introduce beneficial bacteria into the gut, further supporting its health and resilience. In addition, kefir microbiota seems to produce antimicrobial metabolites such as bacteriocins, which inhibit intestinal pathogens [90]. Therefore, a nutrient-dense diet not only enhances overall health but also fortifies the gut microbiota, providing a viral defense against viral infections. These findings are supported by several clinical studies. Particularly, Pu et al. performed a randomized controlled trial in which the participants received a daily oral dose of 300 mL of yogurt supplemented with the probiotic strain *Lactobacillus paracasei* N1115 (N1115) at a concentration of 3.6 × 10^7^ CFU/mL for 12 [91]. The control group retained their normal diet without any probiotic supplementation. The results showed that compared to the control group, the number of individuals diagnosed with an acute URTI and the number of URTI events significantly decreased in the intervention group. The change in the percentage of CD3+ cells in the intervention group was significantly higher than in the control group, suggesting an anti-infective effect of probiotics [91]. Similarly, Shida et al. performed a randomized controlled trial demonstrating the efficacy of fermented milk with Lactobacillus casei in preventing upper respiratory tract infections (URTIs). In this study, 96 eligible male workers aged 30–49 years consumed LcS-FM containing 1.0 × 10^11^ viable LcS cells or control milk (CM) once daily for 12 weeks during the winter season. The incidence of URTIs during the intervention period was significantly lower in the LcS-FM group than in the control group [92].

## 5. Conclusions and Future Perspectives

Intestinal dysbiosis represents a significant but underexplored avenue in studying viral pathogenesis. It is well known that dietary habits can deeply influence the gut microbiota composition, giving it specific immunomodulatory properties. Emerging evidence about the antiviral effects of specific dietary compounds, as illustrated in the present review, suggests the crucial role of a multidisciplinary approach in the prevention of the growing spread of viral infections, as well as in early treatment, as it would make a more rapid response to these pathogens. On the contrary, the wide spread of the Western diet model in the general population could prove to be a strongly predisposing factor for viral infections and related complications.

Moreover, by deepening our understanding of how microbial imbalances affect viral infections, we can develop more comprehensive strategies for prevention and treatment, also involving the nutritional approach. Investigating the complex interactions between the microbiome and viral pathogens holds the potential to unlock new preventive and therapeutic avenues, which could revolutionize our approach to managing viral diseases. 

Interestingly, the gut microbiota competes with pathogens for resources and produces antiviral compounds, directly inhibiting viral replication. Evidence from studies on viruses, such as influenza and rotavirus, demonstrates the protective effects of a healthy gut microbiota. Future research should emphasize longitudinal studies to unravel the causal relationships between dietary habits, dysbiosis, and viral infection outcomes. Such studies will help clarify how shifts in the microbiome influence the severity and progression of infections over time. 

Understanding these interactions opens avenues for therapeutic interventions, including probiotics, prebiotics, and dietary modifications, to enhance the immune system’s ability to contract viral infections. Additionally, there is substantial promise in developing personalized microbiome interventions. Tailoring treatments to individual microbiome profiles could optimize outcomes, making therapy more effective and reducing side effects. Furthermore, integrating microbiome modulation with traditional antiviral therapies could enhance the efficacy of existing treatments, offering a dual approach that leverages the strengths of both microbiological and pharmacological strategies. The future of viral infection management could very well depend on our ability to manipulate the microbiome to support the body’s natural defenses and to enhance the effectiveness of antiviral drugs. As such, the integration of microbiome research into the broader context of viral pathology is not just promising but essential. Despite promising findings, further research is needed to fully elucidate the complex relationships between the gut microbiota and the immune system and to develop effective microbiota-based strategies for viral infection prevention.
